# Application of next-generation sequencing technologies in virology

**DOI:** 10.1099/vir.0.043182-0

**Published:** 2012-09

**Authors:** Alan D. Radford, David Chapman, Linda Dixon, Julian Chantrey, Alistair C. Darby, Neil Hall

**Affiliations:** 1 University of Liverpool, Institute of Infection and Global Health, Leahurst Campus, Chester High Road, Neston, South Wirral CH64 7TE, UK; 2 Institute for Animal Health, Pirbright Laboratory, Ash Road, Pirbright, Woking, Surrey GU24 0NF, UK; 3 University of Liverpool, School of Veterinary Science, Leahurst Campus, Chester High Road, Neston, South Wirral CH64 7TE, UK; 4 Institute of Integrative Biology, Biosciences Building, Crown Street, University of Liverpool, Liverpool L69 7ZB, UK

## Abstract

The progress of science is punctuated by the advent of revolutionary technologies that provide new ways and scales to formulate scientific questions and advance knowledge. Following on from electron microscopy, cell culture and PCR, next-generation sequencing is one of these methodologies that is now changing the way that we understand viruses, particularly in the areas of genome sequencing, evolution, ecology, discovery and transcriptomics. Possibilities for these methodologies are only limited by our scientific imagination and, to some extent, by their cost, which has restricted their use to relatively small numbers of samples. Challenges remain, including the storage and analysis of the large amounts of data generated. As the chemistries employed mature, costs will decrease. In addition, improved methods for analysis will become available, opening yet further applications in virology including routine diagnostic work on individuals, and new understanding of the interaction between viral and host transcriptomes. An exciting era of viral exploration has begun, and will set us new challenges to understand the role of newly discovered viral diversity in both disease and health.

## Introduction

**Fig. 1.**
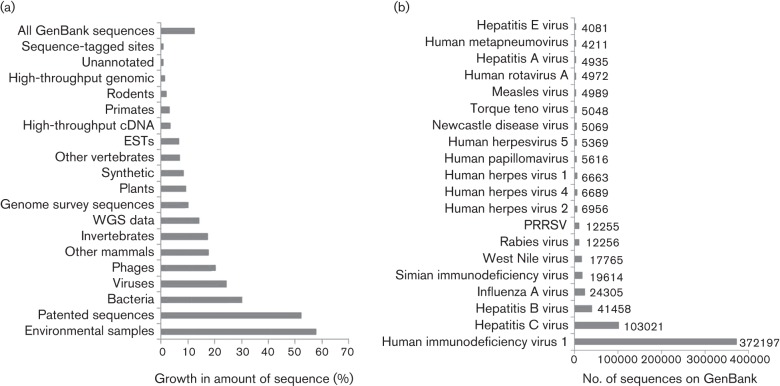
Scale of sequencing information available in GenBank. (a) Percentage growth of sequences deposited on GenBank between August 2009 and August 2010. Sequences are grouped based on the GenBank classification of division, either by their higher taxonomy or the sequencing method employed (Benson *et al.*, 2011). (b) The 20 most frequently sequenced viruses appearing on GenBank. Sequences were identified based on their organism name as described in each submission; PRRSV, porcine reproductive and respiratory syndrome virus. These data were compiled using eid2 (Anonymous, 2012).

We need only to look at the size and growth of international nucleotide databases to realize the importance of sequencing to science in general, and virology in particular. In the latest published release of GenBank (#179; August 2010), there were some 970 million and 43 million bases of viral and phage origin respectively, representing an annual growth of 20–24 % (Fig. 1a) (Benson *et al.*, 2011), a growth rate comfortably above average for the database as a whole. The sequencing effort is being driven almost entirely by human clinical significance, with 17 of the top 20 sequenced viruses causing disease in humans (Fig. 1b). In its entirety, this sequencing effort represents a significant achievement; the information generated has wide-ranging impact on all areas of virology, from diagnosis to pathogenesis, and from vaccine design to viral evolution and ecology.

All of this output has only been possible through embracing what were, at their outset, two groundbreaking and revolutionary methodologies. These allowed those interested in understanding viruses first to amplify and then to sequence viral nucleic acids, namely DNA amplification by PCR (Mullis *et al.*, 1986) and DNA sequencing with chain-terminating inhibitors (Sanger sequencing or first-generation sequencing) (Sanger *et al.*, 1977). Using these technologies, even a fairly basic laboratory could amplify and generate 100–1000 bases of sequence relatively easily in a single day, and by applying first-generation sequencing technology on a large scale in highly specialized sequencing centres, the first large high-profile genomes were published, notably those for *Homo sapiens*, *Plasmodium falciparum* and *Mycobacterium tuberculosis* (Cole *et al.*, 1998; Gardner *et al.*, 2002; Lander *et al.*, 2001). The main limitations to the use of these technologies are restricted scalability, their cost when applied to large genomes and their frequent reliance on prior and specific template amplification by PCR or in bacterial clones. As such, genome projects were largely restricted to high-profile organisms, model organisms and human pathogens.

With these limitations in mind, new methods of sequencing have now been developed (second-generation or, more commonly, next-generation sequencing; NGS) that have entirely revolutionized our ability to sequence. Overnight, it is now possible to generate many millions of bases of sequence and this has opened many new opportunities, making large-scale sequencing projects accessible to us all, whatever our field of biology. In this review, we shall give an overview of these methods and look particularly at their increasing application in virology, specifically in virus discovery, genome sequencing and transcriptomics.

## Technology

There are an increasing number of NGS technologies in the marketplace, all using slightly different methodologies to achieve clonal amplification and sequencing (Table 1). These methodologies are likely to be subject to continual modification, although the basic principles will probably remain the same.

**Table 1.** Summary of current next-generation technologies: methods used in sample preparation, molecule separation and sequencing, and advertised outputs First step for all methods is template fragmentation.

Method	Adapter type	Amplification?	Separation	Sequencing chemistry	Approximate read length (bases)†	Approximate maximum amount of data per run†
Roche 454*	Adapters	Emulsion PCR	Microbeads and ‘picotitre’ plate	Pyrosequencing	400–700	700 Mb
SOLiD	Adapters	Emulsion PCR	Beads on glass slide	Ligation	50–75	20 Gb
Illumina*	Adapters	Bridge amplification *in situ*	Glass slide hybridization	Reversible terminators	25–500	600 Gb
Helicos	Poly(A) adapter	No amplification	Flow-cell hybridization	Reversible terminators	25–55	35 Gb
PacBio	Hairpin adapters	Linear amplification	Captured by DNA polymerase in microcell	Fluorescently labelled dNTPs	1000	Not available
Ion Torrent*	Adapters	Emulsion PCR	Ion Spheres and high-density array	Detection of released H^+^	35–400	1 Gb

* These technologies are available on platforms with different scales of throughput (Loman *et al.*, 2012).

† Approximate values based on data published on the companies’ websites on 9 March 2012. These data are for guidance only and are subject to change; readers interested in the details should consult either the manufacturers or those that are offering the sequencing service.

### Instruments

#### 454 sequencing (Roche Diagnostics).

**Fig. 2.**
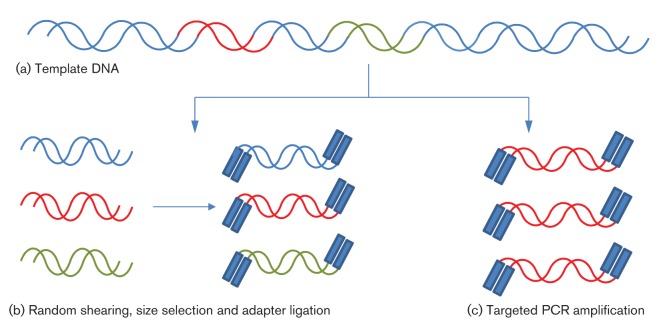
Preparation of DNA. (a) Double-stranded template DNA is (b) randomly degraded, size-fractionated and ligated to adapters. (c) Alternatively, targeted amplification of the template can also be carried out by PCR.

**Fig. 3.**
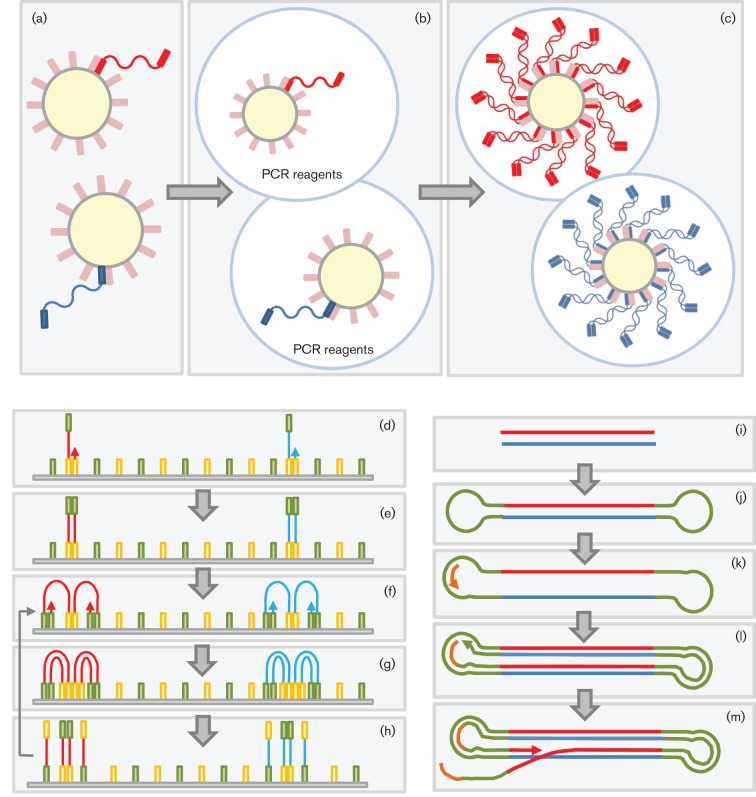
General principles of template amplification. (a–c) Emulsion PCR (Roche 454, SOLiD and Ion Torrent). (a) Adapters are used to capture single molecules of template onto microbeads by primer hybridization. (b) Beads are incorporated into a carefully controlled emulsion, in which each bubble constitutes a microreactor containing DNA template, primer and reagents for PCR. (c) Following amplification, each bead is coated with clonally amplified molecules. (d–h) Bridge amplification (Illumina). (d) Single-stranded template annealed to a glass plate by hybridization to a complementary primer. (e) The primer forms the basis for extension. (f) The free end of each single-stranded molecule can anneal to a second anchored primer in close spatial proximity, forming a ‘bridge’ that acts as a template for (g) a second round of amplification. This results in (h) four linear molecules. Stages (f)–(h) are essentially repeated to generate clonally amplified islands or clusters for subsequent sequencing. (i–m) Linear amplification (PacBio). (i) Template dsDNA. (j) Bound hairpin adapters create a single-stranded circular template. (k) Binding of a primer complementary to hairpin sequence. (l–m) Linear amplification and strand displacement create a single strand of DNA containing multiple copies of plus- and minus-strand sequences that serves as template for sequencing.

**Fig. 4.**
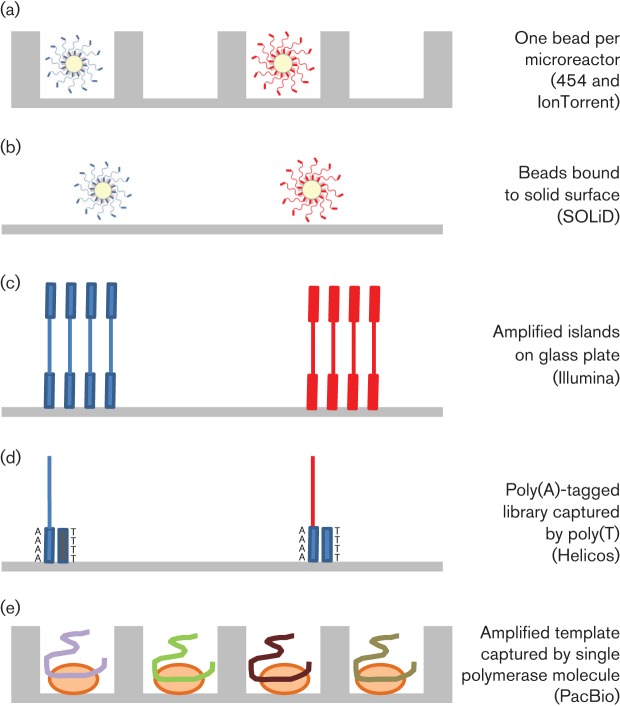
Methods of separating sequencing reactions. (a) Microbeads (Roche 454) or Ion Spheres (Ion Torrent) in microwells. (b) Clonally amplified beads bound to glass plate (SOLiD). (c) Amplified islands (Illumina). (d) Poly(A)-tagged library hybridized to plate (Helicos). (e) Amplified molecule captured by a single DNA polymerase molecule at the bottom of a microcell (PacBio).

**Fig. 5.**
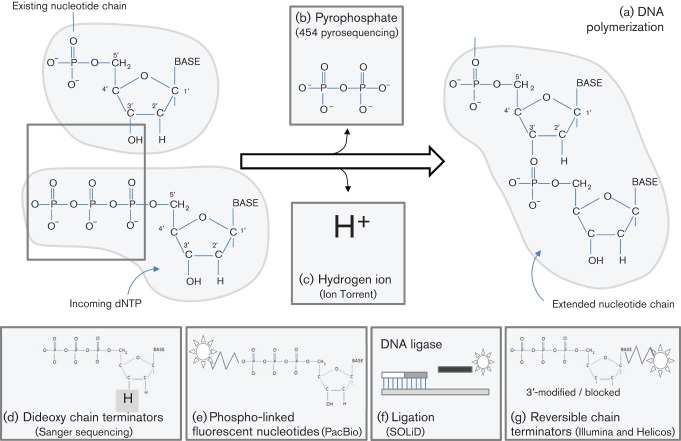
Basic principles of sequencing chemistry. (a) Normal chain elongation leads to the release of (b) pyrophosphate (Roche 454) and (c) a hydrogen ion (Ion Torrent). (d) Structure of the ddNTP that forms the basis of Sanger sequencing. (e) Cartoon of the phospholinked fluorophore (PacBio). (f) Sequencing by ligation (SOLiD – see Fig. 6). (g) Cartoon of reversible chain terminators (Illumina and Helicos).

dsDNA in solution is first sheared by nebulization (converted into a fine spray) or amplified by PCR, and fragments of the appropriate size are selected (Fig. 2). These fragments are blunt-ended and dephosphorylated, followed by the ligation of two separate adapters (A and B). The B adapter is biotinylated, allowing subsequent purification using streptavidin-coated beads. Subsequently, denaturation releases from the beads only those molecules that contain an A primer on one end and a B primer on the other. These molecules constitute the DNA library, which is bound to microbeads through primer hybridization under conditions that favour a single molecule per bead (Fig. 3a). Subsequently, an emulsion is formed in a water and oil mixture, thus capturing individual beads and amplification reagents, including primers, one of which is biotinylated, in their own emulsion microreactor (Fig. 3b). Thermal cycling then results in the emulsion-based clonal amplification of individual molecules (Fig. 3c). After amplification, the emulsion is disrupted and beads coated with amplified products are enriched using streptavidin-coated beads and magnetic separation. The amplified products are denatured, bound to a sequencing primer and separated into individual wells of a picotitre plate, each well being large enough to accommodate a single bead (Fig. 4a). Sequencing then takes place by pyrosequencing, whereby each nucleotide incorporation leads to the release of pyrophosphate (PP_i_) (Fig. 5b). This is converted via ATP to generate light, the amount of which is proportional to the number of bases incorporated. The strength of this system is its read length of approximately 500 bases, making it particularly suitable for amplicon sequencing and bridging across complex sequences. Its Achilles’ heel is its reliance on pyrosequencing, which creates difficulties for sequencing homopolymer runs, leading to an error rate in individual reads of ≤1 %, mostly attributable to insertions and deletions (Gilles *et al.*, 2011; Wang *et al.*, 2007). Whilst depth of coverage can usually overcome this, it becomes more problematic when individual read data are used to assess population diversity (see subsection on Targeted amplification).

#### SOLiD sequencing (Life Technologies).

**Fig. 6.**
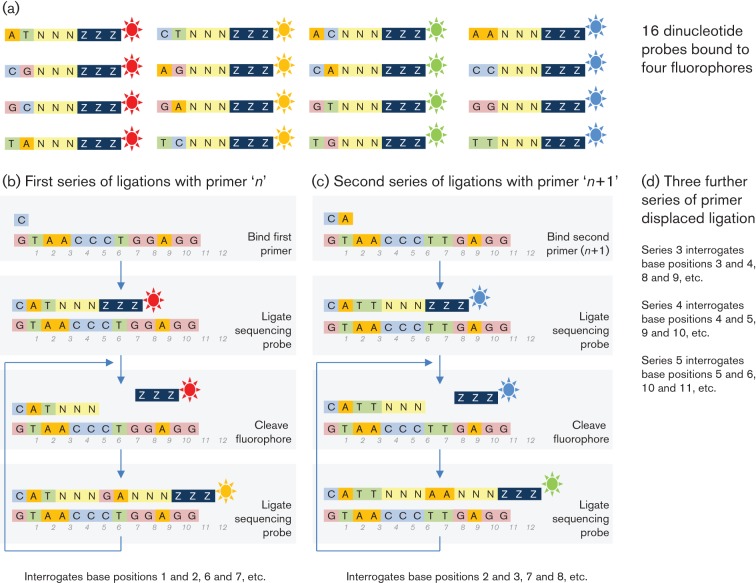
Basic principles underlying sequencing by ligation (synthesis) used by SOLiD.

This methodology starts in a similar way to that described above, with DNA fragmentation (Fig. 2), ligation to beads, and emulsion PCR (Fig. 3a–c). Following denaturation of amplified products, the beads are attached to a glass slide for sequencing (Fig. 4b). The density of beads can be extremely high, and determines the ultimate number of reads achieved. Unlike all other methodologies, sequencing occurs by hybridization and ligation (Fig. 5f) with fluorescently labelled 8mers, which are of the general sequence 3′-CTNNNZZZ-5′–label, where N represents a degenerate base, and Z a ‘universal’ modified base with no binding preference (Fig. 6). Specificity of ligation and sequencing comes only from the 3′ dinucleotides, of which there are clearly 16 combinations. These 16 sequencing probes are labelled with one of four dyes, leaving any individual dye associated with four primers. Sequencing starts by hybridization of a primer and proceeds by the progressive ligation of 8mers, fluorescence detection to identify which sequencing probe pool was incorporated, and cleavage to remove the label and three 5′ universal bases. This leaves a 5mer in place on the newly extended strand, from which the process is repeated. This first round of ligation reactions gathers sequence information on positions 1 and 2, 6 and 7, 11 and 12, etc. This process is then repeated with a starting primer that is displaced by 1 nt in the 5′ direction (upstream), allowing sequence information to be gathered on positions 2 and 3, 7 and 8, 12 and 13, etc. The process is repeated from starting primers displaced 2, 3 and 4 nt upstream. Independent ligation reactions starting from each of these five primers allows the software to recreate the final sequence. It also means that each position in the sequence is interrogated twice, leading to high accuracy.

#### Illumina sequencing.

Following DNA fragmentation, adapter ligation and gel purification, adapter-ligated, single-stranded sequences are annealed to a glass plate that is precoated with oligos complementary to the adapters (Fig. 3d–h). These oligos serve both to capture the template DNA and as primers for subsequent amplification. Amplification occurs on the slide by a process termed ‘bridge amplification’, in which each single-stranded molecule binds at both ends to the oligo primers on the slide. Successive rounds of PCR result in the generation of tiny islands or clusters of amplified molecules (Fig. 3h), which serve as clones for subsequent sequencing using chain terminators, similar to traditional Sanger sequencing. However, unlike the Sanger method, Illumina uses fluorescently labelled reversible terminators, such that each single base incorporation on each molecule temporarily terminates the reaction (Fig. 5g). A high-resolution digital image is used to determine which nucleotide is incorporated in each DNA clonal cluster. After imaging, the terminator is reversed chemically, allowing the template molecule to be extended again in the next round of sequencing. As such, the sequencing reaction proceeds to conclusion on the majority of molecules.

#### Helicos sequencing (Helicos Biosciences).

Helicos has been the first true single-molecule sequencing technology to enter the marketplace, with no template amplification prior to sequencing. The methodology starts in a similar way to those already described, with template fragmentation and adapter ligation (Fig. 2). This time, a poly(A) adapter is added to the 3′ end of the single-stranded DNA template, finishing with a single fluorescently labelled dATP. This poly(A) tag is used to capture each template molecule onto a flow cell using oligo(dT) probes, at a density of over 100 000 000 cm^−2^ (Fig. 4d). A laser locates the bound templates, prior to the cleavage of the 3′ fluorescent label. A DNA polymerase and fluorescently labelled, reversible terminator nucleotides are then added sequentially, with imaging detecting each incorporated base (Fig. 5g). This method has been used to sequence RNA directly without prior cDNA synthesis (Ozsolak *et al.*, 2009).

#### PacBio sequencing (Pacific Biosciences).

Unlike other NGS technologies, in this method it is the DNA polymerase that is immobilized on the floor of a microcell. Fragmented dsDNA is ligated to hairpin adapters to create circular DNA (Fig. 3i–m). These are amplified linearly using primers complementary to hairpin sequence, and then captured by a single molecule of DNA polymerase and sequenced in the bottom of a well (‘Zero Mode Waveguides’) (Fig. 4e). Fluorescently labelled nucleotides (one colour for each base) diffuse into the cell from above. Unlike other systems, the fluorescent molecule is phospholinked, meaning that it is cleaved on incorporation (Fig. 5e). As these labelled nucleotides diffuse around the polymerase active site, they generate a small noise signal. However, when the DNA polymerase encounters the nucleotide complementary to the next base in the template, it is incorporated into the growing DNA chain, and held in place for orders of magnitude longer than the average diffusing nucleotide. This creates a measurable coloured signal that can be differentiated from simple diffusion. Following incorporation, the fluorescent label is cleaved (as part of the chemistry of forming the phosphate chain) and diffuses away, allowing the DNA polymerase to continue to incorporate multiple bases per second.

#### Ion Torrent (Life Technologies).

As with other methodologies, the first stage of Ion Torrent sequencing relies on adding adapters of known sequence to template dsDNA by ligation or PCR. These adapters are used to capture the library clonally onto solid particles, and then to amplify the target sequences through emulsion PCR (Fig. 3a–c). The amplified library is then separated, one bead per well, on a high-density array (Fig. 4a). These wells sit on top of an ion-sensitive semiconductor. During DNA polymerase-catalysed extension, a hydrogen ion is released as part of the normal chemistry of nucleotide incorporation (Fig. 5c). This ion is detected by the semiconductor as a small change in pH. Like pyrosequencing, which measures released PP_i_, the extent of the change in pH with Ion Torrent sequencing is determined by the number of base incorporations, rendering it sensitive to misreading the length of homopolymers.

## Choice of platform

Deciding which technology is best is a contentious issue and will ultimately depend on the specific experiment being planned. Important factors include the size of the genome being considered, its complexity including G+C content, as well as the depth of coverage and accuracy required. It is therefore most important to take advice from local service providers. That said, some general principles based on current technology performance may help to guide the decision-making process. For those looking to assemble complex genomes *de novo*, longer read lengths may be appropriate. For those seeking faster turnaround times, then the smaller platforms may offer greater flexibility, depending on the size of the laboratory (Loman *et al.*, 2012). 454 sequencing (Roche), whilst currently relatively expensive, still has a niche in amplicon sequencing because of its longer reads. Other platforms may be more appropriate for direct RNA sequencing (Helicos) and very long reads (PacBio). However, as both are single-molecule sequencers, accuracy may become an important issue depending on the experiment. In the authors’ experience, the Illumina and SOLiD platforms currently offer the best all-round value for money, accuracy and throughput for RNA-seq (see subsection on Transcriptomics), and those projects requiring high depths of coverage.

## Sample preparation

The amount of sequence generated by each platform may seem excessive for viral genomes that typically range in size from 5 to 350 kb. However, in most cases sequencing occurs on a solid platform, whether a glass or microwell plate. These surfaces can be readily partitioned to allow several samples to be sequenced independently on a single run of the machine. In addition, by the use of distinct adapters that contain unique sequence motifs or tags that are also sequenced during the reaction, libraries of different samples can be mixed and sequenced together, their sequences being partitioned back to their respective samples based on the unique sequence tags in their adapters. This process is usually referred to as barcoding. In addition, many of the companies that manufacture the platforms discussed above are producing smaller machines with such users in mind.

One of the crucial features for ultimate sequencing success is how the template nucleic acid is prepared. Clearly, the appropriate nucleic acid must be purified, whether it be RNA or DNA. As obligate intracellular organisms, viral preparations are usually heavily contaminated by host nucleic acid, and it is wise to remove as much of this as is practical in order to ensure as many of the resulting sequence reads are of viral rather than host origin. For DNA and RNA viruses, the nature of contaminating nucleic acid may vary. In all cases, the best starting material contains little other genetic information, such as cerebrospinal fluid and serum. However, methods are also described for more cellular material (Daly *et al.*, 2011). In many cases, simple viral purification can work: low-speed centrifugation to remove cellular debris, followed by high-speed sedimentation to concentrate packaged viral genomes. RNase and/or DNase treatment can be used to remove unpackaged and unprotected contaminating nucleic acids. In other cases, more specific purification may be followed based on density gradients. Where the goal is to sequence one or just a small number of genomes, crude purification is often sufficient, especially where the academic value of the genomes is high. Under these circumstances, only a small percentage (1 % or less) of the reads would typically be of viral origin. If the goal is to sequence many hundreds of genomes, and where it is important to achieve full genome coverage, then simple methods of prior virus enrichment, including PCR, are needed. More recently, hybridization capture has been used to enrich viral nucleic acid prior to deep sequencing, allowing whole herpesvirus genomes to be sequenced from clinical samples (Depledge *et al.*, 2011). In each experiment, there is a clear trade-off between sample purity and time/cost for purification, and each project needs assessment on its own values.

## Bioinformatics

The informatics requirements of NGS projects are ignored at the researcher’s peril. Arguably, it is easy to now generate many million bases of sequence; the challenge can be to use it efficiently. A full discussion of informatics is beyond the scope of this article. However, key processes in the pipeline are quality scoring, sequence assembly and annotation. Manipulating typical output files, including both individual reads and their alignments into continuous sequences (contigs), can be challenging due to their size.

Post-sequencing, there are two approaches that can be used in analysis of read data. If a reference genome is available, then the sequences can be mapped directly to this reference using a mapper such as bwa (Li & Durbin, 2009). This provides rapid information about substitutions, insertions, deletions and gene loss, but is not applicable if the aim of the research is to look for novel genes or sequence. Under these circumstances, or where a suitable reference genome is not available, individual sequencing reads can be assembled *de novo* using other software such as Velvet (Zerbino & Birney, 2008) or mira (Chevreux *et al.*, 1999), which use algorithms to find overlapping information between reads, leading to the generation of contigs. This approach allows discovery of novel genes and sequences, as well as small variants (e.g. SNPs and indels).

Both the *de novo* and mapping strategies may not cover the entire genome, due either to insufficient depth of coverage or to genomic repeats that, if the read length is smaller than the repeat, cause gaps in the genome assembly. The size of these gaps can be approximated by comparison with a reference genome or, alternatively, may be crossed using paired-end (or mate-paired) libraries. Here, larger fragments of DNA are first purified (e.g. 2–8 kb), adapters are added on either end and the molecule is circularized. This brings two regions of the genome, previously separated by several thousands of bases, into close contact, separated by the adapters. These circular DNA molecules are fragmented and sequenced in the usual way. This not only allows the original ends of the molecule to be sequenced, but crucially also identifies their relative position and separation in the genome. Sequence from paired-end libraries can be built informatically into genome ‘scaffolds’, showing the order and relative positions of individual contigs, thereby facilitating genome closure. Genome gaps can be closed by conventional PCR followed by more traditional Sanger sequencing; whether this is necessary depends on the nature of the project.

## Utilities in virology

### Genome sequencing

Perhaps the most obvious application of these technologies is genome sequencing. Although viral genomes are relatively small, their academic value is often extremely high, and, when coupled with barcoding and partitioning, NGS can represent a highly efficient way of sequencing full viral genomes. It is impractical to provide an exhaustive list of the viral genomes published by NGS, and any such list would rapidly go out of date. Rather, we provide two examples of this approach, chosen from extremes of the viral landscape, namely Marseillevirus, a DNA virus with one of the largest viral genomes known, and influenza virus, a small, segmented RNA virus.

Marseillevirus belongs to the nucleocytoplasmic large DNA viruses (NCLDVs), which includes poxviruses, asfarviruses, iridoviruses, phycodnaviruses and mimiviruses – the biggest type of virus. The genome is a circular dsDNA molecule of 368 454 bp (Boyer *et al.*, 2009). Despite its size, the whole genome sequence was achieved in a single experiment (F. Ghislain, personal communication). In total, 457 ORFs were predicted to encode proteins ranging from 50 to 1537 aa. The coding sequences represented 89 % of the genome. The genome repertoire of the virus was composed of typical NCLDV core genes, as well as genes apparently obtained from eukaryotic hosts and their parasites or symbionts, both bacterial and viral. This study led the authors to propose that amoebae are ‘melting pots’ of microbial evolution where diverse forms emerge, including giant viruses with complex gene repertoires of various origins. A powerful way to compare family members of large genomes is by comparing the ancestral gene set, i.e. the proportion of a genome’s coding capacity that is conserved with other members of the family. Of the 41 NCLDV genes previously identified as comprising the ancestral gene set of the NCLDV (e.g. replication machinery, RNA polymerase subunits, transcription factors, capping and polyadenylation enzymes, DNA-packaging apparatus and structural components of an icosahedral capsid and the viral membrane), only 28 were identified in the Marseillevirus genome, suggesting that whilst Marseillevirus is likely to be a true NCLDV, it is related somewhat distantly to other known virus families.

Almost at the extreme other end of viral biodiversity from Marseillevirus are the influenza viruses, with relatively small, segmented RNA genomes. The need for genome sequencing of these viruses has been driven by the emergence of H5N1 (Höper *et al.*, 2009) and an H1N1 pandemic, with an urgent need to understand the evolution and molecular epidemiology of these viruses. Added sequencing complexity comes from a segmented genome and associated recombination events, meaning that phylogenetic analyses based on partial sequence information can never pick up the full complexity of historical recombination events that often lead to pandemic emergence. Clearly, full genome sequences can be obtained from clinical material by segment-specific PCR and more traditional Sanger sequencing (Smith *et al.*, 2009; Vijaykrishna *et al.*, 2011). However, more recently, methods have been described based on pooling of segment-specific PCRs obtained directly from clinical material (Höper *et al.*, 2009, 2011) or following purification of virus particles from cell culture (Lorusso *et al.*, 2011), followed in both cases by tagging and sequencing. By allowing many full genomes to be sequenced, NGS has effectively removed a major bottleneck to our understanding of the emergence and transmission of these important viruses (Baillie *et al.*, 2012). The remaining challenge is to reduce the time for sequence production to a point where sequence information can be used routinely in real time to aid health protection agencies in the control of outbreaks.

In the authors’ laboratories, we have now used NGS platforms to obtain *de novo* sequence from multiple virus families, including *Poxviridae*, *Parvoviridae*, *Picornaviridae*, *Herpesviridae*, *Asfarviridae* (Chapman *et al.*, 2011) and *Rhabdoviridae*, underlying the broad applicability of these techniques to this field of virology. Cleary, single full genome sequences are incredibly valuable to understanding virus biology, but perhaps of more interest is the ability that NGS provides to sequence and compare multiple full genomes of distinct types, to identify important genetic differences between them (Szpara *et al.*, 2010).

### Targeted amplification of virus to look at resistance profiles to drugs and host immunity

One of the striking features of viral genomes is their potential for high evolution rates, a feature of short generation times and, for RNA viruses in particular, low-fidelity polymerases. This has for many years been enshrined in the concept of the viral quasispecies, whereby many RNA viruses are believed to exist at the sample level, not as a single sequence but as a collection of closely related variants (Lauring & Andino, 2010). This diversity creates a challenge for those wishing to sequence viruses, such that many of the sequences for RNA viruses, particularly those based on PCR without cloning, represent average, majority or consensus sequences for these populations. Generally, minority members of the population are ignored, which in most cases is entirely acceptable practice. However, in evolutionary terms, this existing diversity represents a massive reserve on which selection can occur and from which fitter variants can emerge rapidly. These are of concern not only for immune escape, but also in antiviral chemotherapy, where minor population variants harbouring resistance mutations can be rapidly selected for, leading to failure of antiviral therapy. This has led the World Health Organization (WHO) to establish a global strategy for the prevention and assessment of human immunodeficiency virus (HIV) drug resistance and, amongst its many roles, the Global Influenza Surveillance and Response System has been closely monitoring the evolution of influenza viruses infecting humans, including their susceptibility to antiviral drugs.

There is a long history of attempts to characterize the viral quasispecies and to identify clinically significant minor variants within it. Generally, this has been done by PCR amplification and direct sequencing, which may only be able to detect mutations that exist at >10–20 % frequency in the population (Varghese *et al.*, 2009; Wang *et al.*, 2007), or line probe assays, which, although perhaps slightly more sensitive, can only detect known mutations (Lok *et al.*, 2007). Sensitivity can be increased by using limiting-dilution PCR, i.e. essentially sequencing multiple individual molecules from each sample; however, this clearly comes at a considerable cost (Palmer *et al.*, 2005; Wang *et al.*, 2007). In comparison, it is generally accepted that NGS of amplicons can detect minority variants present in 1–2 % of sequence reads (Varghese *et al.*, 2009), and it is now accepted that NGS detects many more minority mutations than more traditional methods (Margeridon-Thermet *et al.*, 2009). For example, in one study of HIV population mutations, ultradeep pyrosequencing detected on average seven times more variants than conventional methods, with all variants present in 3 % of genomes and 57 % of variants present in <3 % of genomes confirmed by limiting-dilution sequencing (Wang *et al.*, 2007). The limitation to this detection threshold is driven both by error rates of the polymerases used and by pyrosequencing errors around homopolymers, and has been estimated by amplifying plasmid sequences (Margeridon-Thermet *et al.*, 2009; Mitsuya *et al.*, 2008; Solmone *et al.*, 2009; Varghese *et al.*, 2009; Wang *et al.*, 2007). To the authors’ knowledge, error rates associated with reverse transcription have not been included, so this represents an important area for future research, particularly as direct RNA sequencing becomes available (Ozsolak *et al.*, 2009).

NGS has predominantly been used to monitor population diversity in HIV (Eriksson *et al.*, 2008; Hoffmann *et al.*, 2007; Ji *et al.*, 2010; Wang *et al.*, 2007) and hepatitis B virus (Homs *et al.*, 2011; Margeridon-Thermet *et al.*, 2009; Solmone *et al.*, 2009), but it is now starting to be used for other viruses (Geret *et al.*, 2011; Görzer *et al.*, 2010; Hiraga *et al.*, 2011; Tapparel *et al.*, 2011; Verbinnen *et al.*, 2010; Wellehan *et al.*, 2010). The methodology is starting to shed new light on other areas of viral pathogenesis where minor population variants may also be highly significant. These include assays for receptor usage (Raymond *et al.*, 2011) and minor pathogenic variants (Geret *et al.*, 2011), and studies to understand immune escape in simian immunodeficiency virus (Bimber *et al.*, 2009; Hughes *et al.*, 2010). Studies using NGS to dissect the dynamics of transmission have shed new light on the differences between resistance acquired by transmission and that which has evolved within the patient (Varghese *et al.*, 2009). In addition, some studies are now moving away from sequencing of specific genomic regions to estimating the diversity across the whole genome (Bimber *et al.*, 2010; Tapparel *et al.*, 2011; Willerth *et al.*, 2010). Such an approach is likely to generate new insights into the roles of previously understudied viral genes in pathogenesis. The use of barcoding and multiplexing provides opportunities to increase the cost-effectiveness of this approach (Hoffmann *et al.*, 2007; Ji *et al.*, 2010).

### Viral metagenomics

Metagenomics can be defined as the characterization of genetic information directly from samples. It has the benefit of not requiring previous culture, making it particularly attractive when trying to characterize the ‘viral metagenome’ or ‘virome’. The term ‘metagenome’ first appeared in PubMed in 1998 (Handelsman *et al.*, 1998) in relation to classifying unculturable bacteria from soil, when such early studies relied on the use of random bacterial cloning and sequencing (Breitbart *et al.*, 2003). The availability of NGS has allowed these metagenomes to be analysed in unbiased ways at previously unseen resolution, and is providing a wealth of new opportunities in two major areas: viral candidate pathogen discovery and viral ecology.

#### Virus candidate pathogen discovery.

The threat of newly emerging viruses to important species remains ever-present. In humans, although viruses currently make up a small proportion of known human diseases, they make up the majority of newly identified ones (Woolhouse & Gaunt, 2007). The rate of discovery of new virus infections has remained largely unchanged over many decades and suggests that many new viruses remain to be discovered (Woolhouse *et al.*, 2008).

In the face of new outbreaks of a disease, there is an imperative to rapidly characterize the infectious agent to understand better the disease, and to allow the development of specific diagnostic tests and control measures. Ideal methods are non-specific and have included electron microscopy, virus isolation, cloning, degenerate or consensus PCR, representational difference analysis (RDA) and sequence-independent single primer amplification (SISPA) (Ambrose & Clewley, 2006; Bexfield & Kellam, 2011; Tang & Chiu, 2010). The most high-profile recent example of this was the SARS (severe acute respiratory syndrome) coronavirus. The new respiratory syndrome was first reported by the WHO on 14 March 2003(Anonymous, 2003). Initially, large-scale screens for known pathogens were carried out to try to identify a common factor in cases using virus isolation, electron microscopy, PCR and antigen-based methods (Drosten *et al.*, 2003). Several ‘red herrings’ were identified, most notably a paramyxovirus. Ultimately, random PCR (Drosten *et al.*, 2003) or degenerative coronavirus primers (Ksiazek *et al.*, 2003) amplified the first SARS coronavirus sequences, which were published electronically in the *New England Journal of Medicine* on 10 April 2003. On 5 July 2003, the WHO declared the SARS outbreak contained (http://www.who.int/mediacentre/news/releases/2003/pr56/en/).

Whilst the SARS story represented a considerable triumph in global cooperation and pathogen discovery, with only approximately 1 month between syndrome description and pathogen discovery, it is highly likely that any such future discovery projects would be carried out by NGS in a fraction of the time, and here we describe just one of many examples that shows the speed of such approaches. In 2008, an outbreak of unexplained haemorrhagic fever in people was reported in South Africa. The index patient was admitted to a clinic on 12 September 2008. Over the following 2 weeks, secondary and tertiary cases were reported, with four of the five patients dying. Such outbreaks of haemorrhagic fever are highly emotive events, necessitating a rapid response to control both infection and public anxiety. RNA extracts from two post-mortem liver biopsies (cases 2 and 3) and one serum sample (case 2) were submitted to NGS. blast analysis of the resulting sequences identified contigs corresponding to about half of the approximately 10 kb genome of a novel arenavirus (Briese *et al.*, 2009). The majority of sequences were obtained from serum rather than tissue, presumably reflecting the higher levels of host DNA obtained from the highly cellular tissue samples.

Such rapid results are highly significant for several reasons. As the method used is not predicated on sequence-specific amplification, it has a high probability of success, providing that sufficient sequencing is done. Indeed, for influenza virus it has been shown that NGS has sensitivity on clinical specimens close to that of specific PCR (Greninger *et al.*, 2010). Secondly, rapid pathogen discovery is now accessible to all outbreaks, regardless of the profile of the disease, as reported recently for Schmallenberg virus of ruminants (Hoffmann *et al.*, 2012). Thirdly, it works very quickly, allowing authors to obtain a candidate diagnosis in only 72 h. Finally, when used in novel samples, it often finds a wealth of previously unrecognized viruses (Day *et al.*, 2010).

This last point highlights a key issue: clearly, the identification of viral sequences in clinical material is far from showing an association with disease (Lipkin, 2009). In some cases, small case–control studies have sought to compare metagenomes in health and disease, including in cystic fibrosis (Willner *et al.*, 2009) and chronic fatigue syndrome (Sullivan *et al.*, 2011), to try to determine the significance of any one genome to disease. However, cost will currently limit such an approach to those diseases where a single pathogen is perhaps unlikely. More typically, traditional methods will be employed to seek proof of an association between any newly discovered virus and disease. These methods include classical infection studies to achieve Koch’s postulates, larger case–control studies using traditional diagnostics methods such as PCR to compare the prevalence of the new viral sequences in health and disease, or the detection of acquired immunity showing a temporal association between infection and disease. Even when all this is done, it is likely that NGS technologies will lead to the identification of an expanding class of viruses that historically would have been called ‘orphan’ viruses, viruses in search of a disease, and commensals (Dolgin, 2011). The latter represents an exciting area of research. For obvious reasons, much of virology has historically been driven by those viruses responsible for disease. Technologies such as NGS may now allow us to open a window on the role of viruses in our health.

#### Environmental viral metagenomics.

Moving away from pathogen discovery in clinical samples, NGS is being used increasingly to explore the viral diversity in a wide range of environmental samples, shedding new light on what might be considered normal.

Recent examples where this technology is being applied include faeces (Donaldson *et al.*, 2010; Li *et al.*, 2010; Reyes *et al.*, 2010), sewage (Cantalupo *et al.*, 2011), vaccines (Victoria *et al.*, 2010), plants (notably grapevines) (Coetzee *et al.*, 2010) and environmental samples including water (López-Bueno *et al.*, 2009; Rodriguez-Brito *et al.*, 2010). A recent paper has described the use of blood-feeding mosquitos as a way of sampling a broad range of viral diversity (vector-enabled metagenomics) and of identifying a broad range of animal, plant, insect and bacterial viruses (Ng *et al.*, 2011).

A review of ocean viromes suggests that the sea is dominated by rare genes, many of which might be contained within virus-like entities such as gene-transfer agents (Kristensen *et al.*, 2010). The same study predicted a wealth of DNA viruses belonging to the eukaryotic NCLDVs, and suggested that the RNA virome was dominated by picorna-like viruses.

Although virus candidate pathogen discovery and environmental viral metagenomics can be broadly thought of as separate disciplines, they are highly related, as they both essentially seek to identify correlations between virus populations and sample phenotype. Recurring themes often include using samples low in contaminating host nucleic acid, such as faeces (Victoria *et al.*, 2009) or serum (Towner *et al.*, 2008), filtration of samples to remove contaminating genetic material, and sequence-independent reverse transcription and PCR amplification of capsid-protected, nuclease-resistant viral nucleic acids. As the wide range of undiscovered viral diversity can now begin to be illuminated, an important challenge is how to identify truly novel sequences when comparing newly generated sequence to published databases. Several approaches have been developed to facilitate this, including searching based on amino acid similarity and by the recreation of theoretical ancestral sequences (Delwart, 2007). As with other molecular methods, the sensitivity of NGS, which can detect rare viral transcripts at frequencies lower than 1 in 1 000 000 (Moore *et al.*, 2011), does bring with it considerable potential for sample contamination (Schmieder & Edwards, 2011).

### Transcriptomics

Whilst genomic data are clearly extremely valuable in their own right, other areas of science are turning increasingly to the transcriptome, the measurement of mRNA, to achieve new insights into genome expression and how this may be modified in health and disease. Historically, oligonucleotide microarrays have been used to quantify gene expression throughout biology, but now these approaches are largely being replaced by NGS of transcribed RNA – so called RNA-seq (Marioni *et al.*, 2008; Wilhelm & Landry, 2009).

Key steps in the process firstly include isolation of RNA and removal of host genomic DNA. Subsequently, rRNA is also removed by selection of polyadenylated RNA or removal of rRNA with antisense oligos. Complementary DNA synthesis is primed either by oligo(dT) or randomly. On long transcripts, the former can lead to bias sequencing of the 3′ end and failure to determine the transcript start site. Strand specificity of transcription is lost in routine preparation of double-stranded cDNA. However, it can be maintained either by adding different adapters to the 5′ and 3′ ends of the RNA transcript, or by marking one strand for degradation by chemical modification (Levin *et al.*, 2010). Analysis of RNA-seq data is usually achieved by mapping sequence reads to a reference genome, using software that can map reads over gene splice junctions (Trapnell *et al.*, 2009). Statistical analysis of the number of reads per genome region (gene) can be used to quantify relative levels of expression (Anders & Huber, 2010; Robinson *et al.*, 2010).

Whilst the use of these technologies is still in its infancy in virology, they are beginning to provide new insight on genome coding capacity and biology of transcription, particularly for large DNA viruses. For example, in mimiviruses, RNA-seq has identified a new role for palindromic sequences in transcription termination (Byrne *et al.*, 2009), confirmed that all predicted ORFs are transcribed, as well as identifying new ORFs (Legendre *et al.*, 2010, 2011). In poxviruses, RNA-seq has been used to revisit temporal trends in gene expression (Yang *et al.*, 2011b), to identify interactions between the host and poxviral transcriptome (Yang *et al.*, 2010) and to map transcription start and stop sites precisely (Yang *et al.*, 2011a). In herpesviruses, new insight has been made into the role of viral gene expression in Epstein–Barr virus latency (Lin *et al.*, 2010), and in cytomegalovirus, the significance of non-coding transcripts and splice variants has been characterized, as well as identifying new protein-coding transcripts (Gatherer *et al.*, 2011). The technology is clearly not limited to large DNA viruses, and has also been used to identify differences in expression and post-transcriptional modification associated with lentivirus virulence (Ertl *et al.*, 2011).

NGS also provides a new way to explore the role of microRNAs in virus replication and pathogenesis. These short RNA molecules of about 21–22 bases in length function as negative regulators of mRNA translation (Sayed & Abdellatif, 2011) and their role is now being explored in several viral systems, including latency in herpes simplex virus (Umbach *et al.*, 2008) and Epstein–Barr virus (Riley *et al.*, 2012), cytomegalovirus (Stark *et al.*, 2012), transformation in Marek’s disease virus (Burnside *et al.*, 2006) and Kaposi’s sarcoma-associated herpesvirus (Wu *et al.*, 2011), and grapevine pathology (Pantaleo *et al.*, 2010).

Of course, there are two sides to a transcriptome for viruses, namely that of the virus and its host. Virus-induced perturbations to the host transcriptome are clearly of critical importance to infection outcome. However, methodological limitations have meant that, until recently, the study of pathogenesis has been heavily driven by targeted analysis of specific genes or pathways thought to be critical to disease progression. Although this review has concentrated on the use of NGS to study viral sequences directly, it is clear that these approaches provide new opportunities to explore the interaction of replicating viruses with their host, particularly their transcriptome, in a non-targeted, hypothesis-generating mode. Recent examples include gene profiling of early and advanced liver disease in chronic hepatitis C virus (HCV)-infected patients (Khalid *et al.*, 2011), the replication of HCV *in vitro* (Woodhouse *et al.*, 2010), host gene shut-off in Kaposi’s sarcoma-associated herpesvirus, and the identification of host transcripts refractory to shut-off (Clyde & Glaunsinger, 2011).

## The future

Despite the rapid progress that has been made in these technologies over the last 5 years, new developments are still likely. In the last year, there has been a lot of publicity around single-molecule nanopore technologies that aim to allow simple, cheap, single-molecule DNA sequencing in devices no larger than a smartphone (Eisenstein, 2012). These technologies work by pulling DNA through tiny pores and reading the bases as they pass through as changes in electric current across the pore. This process could remove the need for enzymic processes and imaging completely and could potentially generate very long reads very rapidly. Whilst no substantial data have yet been seen, this method could become available in 2013. If the technology delivers, then devices could be available in clinics very rapidly afterward, becoming a key technology in diagnostic medicine.

As all of the available sequencing chemistries are becoming established and maturing, read lengths and fidelity should increase, allowing us to explore deeper into viral genome diversity. All of the major platforms are being developed to be easier to use and more cost-effective, and some of the major companies have now released cheaper benchtop machines, with the aim of democratizing this technology and making it more readily available (Loman *et al.*, 2012). The ultimate consequence of such democratization will be to bring NGS sequencing into many more research laboratories, as well as routine diagnostics for human, animal and plant disease, thereby providing new insights into the complex virome in which we all live. In medicine, routine sequencing of the human genome will herald new opportunities for personalized medicine. It perhaps follows that similar opportunities allowing antiviral therapy to be better-matched to individual genome sequences will exist, as already happens to some extent for HIV.

Improvements in the area of sample preparation have been rapid, including recent developments in genome partitioning (also known as enrichment). This allows targeting of a specific region, sets of genes or indeed entire exomes. Technology platforms include either microarrays on solid substrates, free RNA or DNA oligos that can be separated from other fragments using biotin tags, or high-throughput PCR-based techniques (reviewed by Mamanova *et al.*, 2010). Such approaches may be particularly appropriate for viruses as a way of removing contaminating host DNA.
